# Application of the Onodera prognostic nutrition index and neutrophil-to-lymphocyte ratio in risk evaluation of postoperative complications in Crohn’s disease

**DOI:** 10.1038/s41598-017-09265-3

**Published:** 2017-08-16

**Authors:** Wei-Ming Kang, Chang-Zhen Zhu, Xiao-Xu Yang, Jian-Chun Yu, Zhi-Qiang Ma, Xin Ye, Kang Li, Dong Liu

**Affiliations:** Department of General Surgery, Peking Union Medical College Hospital, Chinese Academy of Medical Science and Peking Union Medical College, Beijing, 100730 China

## Abstract

This study aimed to investigate application of Onodera prognostic nutrition index (OPNI) and neutrophil-to-lymphocyte ratio (NLR) in evaluating risk of postoperative complications in Crohn’s disease (CD). Clinical data of 108 postoperative CD patients in 9 years were respectively reviewed. OPNI and NLR were within 1 week preoperatively. Average OPNI was 38.8 ± 8.2 and significantly lower in patients with: CD type B3; lymphopenia; decreased haemoglobin, prealbumin, and albumin; and daily enteral nutrition <500 kcal/d. Average NLR was 5.9 ± 12.1 and significantly higher in patients with: CD type B3, neutrophilia, lymphopenia, decreased prealbumin, and enteral nutrition <500 kcal/d. Youden index was maximal at OPNI 39.8 and NLR 4.1, patients were divided into two groups by OPNI 39.8 and NLR 4.1; Low OPNI (≤39.8) group had significantly greater incidence of type B3, lymphopenia, decreased haemoglobin, prealbumin and albumin, and enteral nutrition <500 kcal/day, more likely to have intra-abdominal bleeding. High NLR group (≥4.1) had significantly greater incidence of type B3, neutrophilia, and lymphopenia, more likely to develop lung infection. OPNI and NLR were significantly negatively correlated. Smoking within 1 year preoperatively, OPNI <39.8, NLR ≥ 4.1 were independent risk factors for postoperative complications in CD.

## Introduction

The Onodera prognostic nutrition index (OPNI), established by Onodera after the analysis of 200 gastrointestinal surgery patients in 1984, is an index used to evaluate the nutritional condition and predict surgical risk of gastrointestinal surgery patients^1, 2^. Many studies have investigated the relationship between the OPNI and gastrointestinal diseases^3, 4^. Another parameter used for prognostic evaluation in gastrointestinal diseases is the neutrophil-to-lymphocyte ratio (NLR), which is an index used to measure the severity of systemic inflammation^5, 6^. Crohn’s disease (CD) is an inflammatory gastrointestinal disease that can cause systemic inflammation and severe malnutrition, which influences prognosis and postoperative recovery. Although several studies have reported the relationship between OPNI and postoperative complications, NLR and Crohn’s disease severity in Crohn’s disease patients, no studies, until now, have yet investigated whether OPNI and NLR could be used for evaluation of postoperative complications in Crohn’s disease patients at same time. In the present study, we investigated the application of the OPNI and the NLR in risk evaluation of postoperative complications in patients with CD.

## Results

### Distribution of OPNI and NLR According to Clinical Features

The average OPNI and NLR of all patients were 38.8 ± 8.2 and 5.9 ± 12.1, respectively. The distribution of OPNI and NLR according to clinical features, such as sex, age, smoking history, blood type, primary lesion location, disease type, and preoperative BMI are shown in Tables 1 and 2.

**Table 1:** Distributions of OPNI according to clinical features.

clinical features	cases (n = 108)	OPNI	P	clinical features	cases (n = 108)	OPNI	P
**Gender**			>0.05	**PL**			>0.05
Male	67	38.8 ± 8.0		L1 + L2	50	39.5 ± 9.4	
Female	41	39.8 ± 8.9		L3	58	38.5 ± 7.0	
**Age(y)**			>0.05	**DT**			**<0.05**
≤40(A1 + A2)	62	38.5 ± 8.4		B1 + B2	72	41.2 ± 8.1	
>40(A3)	46	37.5 ± 8.6		B3	36	36.5 ± 7.7	
**SH**			>0.05	**POD (y)**			>0.05
NO	76	39.7 ± 9.2		≤1	60	39.3 ± 8.2	
YES	38	37.9 ± 5.6		1 < n ≤ 3	22	38.3 ± 9.7	
**HA**			>0.05	3 < n ≤ 5	9	38.4 ± 7.1	
NO	83	38.2 ± 8.7		5 < n ≤ 10	15	39.5 ± 7.2	
YES	25	42.6 ± 6.0		>10	2	49.6 ± 11.2	
**EO**			>0.05	**PNC**			>0.05
NO	81	41.5 ± 6.8		Normal	92	38.6 ± 8.5	
YES	27	32.2 ± 8.6		Higher	16	39.7 ± 6.9	
**BT**			>0.05	**PLC**			**<0.05**
A+	27	39.3 ± 7.6		Normal	79	41.3 ± 6.7	
B+	32	38.3 ± 9.6		Lower	29	32.1 ± 8.4	
O+	36	39.3 ± 8.0		**PH**			**<0.05**
AB+	12	41.2 ± 8.1		Normal	31	44.8 ± 7.5	
**EM**			>0.05	Lower	77	36.9 ± 7.6	
NO	81	39.0 ± 9.1		**PPA**			**<0.05**
YES	27	39.7 ± 5.6		Normal	28	45.1 ± 5.3	
**PL**			>0.05	Lower	80	37.1 ± 8.2	
NO	94	38.8 ± 8.6		**PA**			**<0.05**
YES	14	41.8 ± 5.6		Normal	49	45.3 ± 4.3	
**p-BMI**			>0.05	Lower	59	33.4 ± 6.7	
**<**18.5	69	38.9±9.0		**PEN**			**<0.05**
≥18.5	39	38.6 ± 6.6		EN	62	42.7 ± 6.6	
				NO EN	46	35.0 ± 8.7	

**SH**, smoking history; **HA**, history of appendectomy; **POD**, preoperative duration; **PL**, primary lesion; **DT**, disease type (B3 or not); **EM**, extraintestinal manifestations; **PL**, perianal lesions; **EO**, emergency operation; **p-BMI**, preoperative BMI; **BT**, blood type; **PH**, preoperative haemoglobin; **PNC**, preoperative neutrophil count; **PLC**, preoperative lymphocyte count; **PA**, preoperative albumin; **PPA**, preoperative prealbumin; **PEN**, preoperative enteral nutrition.

**Table 2:** Distributions of NLR according to clinical features.

clinical features	cases (n=108)	NLR	P	clinical features	cases (n=108)	NLR	P
**Gender**			>0.05	**PL**			>0.05
Male	67	7.5 ± 13.8		L1+L2	50	5.7 ± 5.6	
Female	41	7.5 ± 8.8		L3	58	9.0 ± 15.6	
**Age(y)**			>0.05	**DT**			**<0.05**
≤40(A1+A2)	62	8.3 ± 14.6		B1+B2	72	6.8 ± 13.7	
>40(A3)	46	6.4 ± 7.5		B3	36	8.8 ± 8.0	
**SH**			>0.05	**POD (y)**			>0.05
NO	70	6.0 ± 5.6		≤1	60	7.1 ± 14.2	
YES	38	10.2 ± 18.8		1<n≤3	22	6.6 ± 7.6	
**HA**			>0.05	3<n≤5	9	10.1 ± 8.9	
NO	83	6.1 ± 6.2		5<n≤10	15	9.1 ± 11.6	
YES	25	11.9 ± 22.3		>10	2	4.9 ± 1.6	
**EO**			>0.05	**PNC**			**<0.05**
NO	81	7.8 ± 13.6		Normal	92	5.0 ± 4.3	
YES	27	6.6 ± 5.6		Higher	16	21.8 ± 26.0	
**BT**			>0.05	**PLC**			**<0.05**
A+	27	4.5 ± 4.0		Normal	79	4.3 ± 3.9	
B+	33	9.2 ± 18.7		Lower	29	16.2 ± 20.3	
O+	36	7.7 ± 9.4		**PH**			>0.05
AB+	12	8.9 ± 7.6		Normal	31	6.6±6.7	
**EM**			>0.05	Lower	77	7.8 ± ± 13.7	
NO	81	6.8 ± 7.6		**PPA**			**<0.05**
YES	27	9.4 ± 20.6		Normal	28	5.0 ± 5.7	
**PL**			>0.05	Lower	80	8.4 ± 13.6	
NO	94	7.1 ± 12.1		**PA**			**>0.05**
YES	14	9.9 ± 12.5		Normal	49	5.4 ± 5.7	
**p-BMI**			>0.05	Lower	59	9.2 ± 15.4	
<18.5	69	8.8 ± 14.7		**PEN**			**<0.05**
≥18.5	39	5.2 ± 4.3		EN	62	5.5 ± 6.4	
				NO EN	46	10.2 ± 16.7	

**SH**, smoking history; **HA**, history of appendectomy; **POD**, preoperative duration; **PL**, primary lesion; **DT**, disease type (B3 or not); **EM**, extraintestinal manifestations; **PL**, perianal lesions; **EO**, emergency operation; **p-BMI**, preoperative BMI; **BT**, blood type; **PH**, preoperative haemoglobin; **PNC**, preoperative neutrophil count; **PLC**, preoperative lymphocyte count; **PA**, preoperative albumin; **PPA**, preoperative prealbumin; **PEN**, preoperative enteral nutrition.

The mean OPNI of the control group was higher than that of the patients with disease type B3 (t = 2.247, *P* = 0.027), with preoperative lymphopenia (t = 5.880, *P* = 0.000), lower than normal haemoglobin (t = 4.984, *P* = 0.000), albumin (t = 11.178, *P* = 0.000), and prealbumin (t = 4.987, *P* = 0.000), and patients whose enteral nutritional intake was less than 500 kcal/day within 2 weeks preoperatively (t = 5.027, *P* = 0.000).

The mean NLR of the control group was lower than that of the patients with disease type B3 (*P* = 0.009), preoperative neutrophilia (*P* = 0.000), preoperative lymphopenia (*P* = 0.000), lower than normal prealbumin (*P* = 0.028), and an enteral nutritional intake of less than 500 kcal/day within 2 weeks preoperatively (*P* = 0.043).

### ROC Curves for OPNI and NLR, and a Comparison of Clinical Features After Grouping According to the OPNI and NLR Cutoff Values

**Figure 1:**
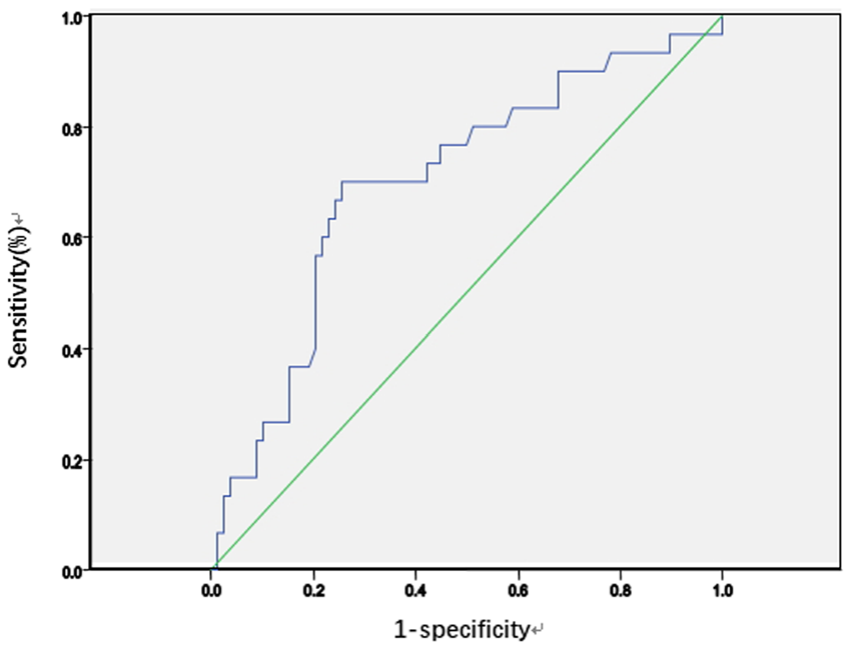
ROC curve of the OPNI.

We drew the ROC curve of the OPNI according to whether postoperative complications occurred (Fig. 1).

The area under the curve was 0.699. When the OPNI was 39.8, the Youden index was maximal, with a sensitivity of 70% and specificity of 68%, and the prediction of risk of postoperative complications was most accurate. Taking 39.8 as the cutoff value, patients were divided into the high OPNI group (OPNI ≥ 39.8, n = 53) and the low OPNI group (OPNI < 39.8, n = 55). The differences in clinical features between the two groups were then analysed (Table 3).

**Table 3:** Clinical features in the high OPNI group (n = 53) versus the low OPNI group (n = 55).

clinical features	h-OPNI G (53 cases)	l-OPNI G (55 cases)	P	clinical features	h-OPNI G	l-OPNI G	P
**Gender**			>0.05	**PL**			>0.05
Male	35	32		L1+L2	27	23	
Female	18	23		L3	26	32	
**Age(y)**			>0.05	**DT**			**<0.05**
≤40(A1+A2)	30	32		B1+B2	41	31	
>40(A3)	23	23		B3	12	24	
**SH**			>0.05	**POD (y)**			>0.05
NO	38	32		≤1	33	27	
YES	15	23		1**<**n≤3	10	12	
**HA**			>0.05	3**<**n≤5	3	6	
NO	43	40		5**<**n≤10	5	10	
YES	10	15		>10	2	0	
**EO**			>0.05	**PNC**			>0.05
NO	39	42		Normal	44	48	
YES	14	13		Higher	9	7	
**BT**			>0.05	**PLC**			**<0.05**
A+	16	11		Normal	46	33	
B+	17	16		Lower	7	22	
O+	6	6		**PH**			**<0.05**
AB+	14	22		Normal	25	6	
**EM**			>0.05	Lower	28	49	
NO	41	40		**PPA**			**<0.05**
YES	12	15		Normal	21	7	
**PL**			>0.05	Lower	32	48	
NO	49	45		**PA**			**<0.05**
YES	4	10		Normal	47	2	
**p-BMI**			>0.05	Lower	6	53	
**<**18.5	37	32		**PEN**			**<0.05**
≥18.5	16	23		EN	42	20	
				NO EN	11	35	

**h-OPNI G**, high OPNI group; **l-OPNI G**, low OPNI group; **SH**, smoking history; **HA**, history of appendectomy; **POD**, preoperative duration; **PL**, primary lesion; **DT**, disease type (B3 or not); **EM**, extraintestinal manifestations; **PL**, perianal lesions; **EO**, emergency operation; **p-BMI**, preoperative BMI; **BT**, blood type; **PH**, preoperative haemoglobin; **PNC**, preoperative neutrophil count; **PLC**, preoperative lymphocyte count; **PA**, preoperative albumin; **PPA**, preoperative prealbumin; **PEN**, preoperative enteral nutrition.

Compared with the high OPNI group, the low OPNI group had a greater incidence of: disease type B3 (X2 = 5.354, *P* = 0.021); preoperative lymphopenia (X2 = 9.864, *P* = 0.002); decreased preoperative haemoglobin (X2 = 17.341, *P* = 0.000), prealbumin (X2 = 10.166, *P* = 0.001), and albumin (X2 = 75.363, *P* = 0.000); and enteral nutritional intake less than 500 kcal/day within 2 weeks preoperatively (X2 = 20.298, *P* = 0.000).

In order to find what specific types of complications can be caused by low OPNI, we calculated the incidence of seven complications in the low and high OPNI group (Table 4) and each complication was listed as fourfold table according to the incidence of complications and the level of OPNI (Table 5). we found that when OPNI was below cutoff value (39.8), Crohn’s disease patients were more likely to have intra-abdominal bleeding (14.55%, P = 0.018).

**Table 4:** The incidence of seven complications in the low and high OPNI group.

OPNI	Total number	low group (n = 55 < 39.8)	high group(n = 53 > 39.8)
Complications		Number (Proportion)	Number (Proportion)
Intraperitoneal haemorrhage	9	8 (14.55%)	1(1.89%)
Wound infection	6	4 (7.27%)	2(3.77%)
Intestinal perforation or fistula	5	3 (12.73%)	2(3.77%)
Respiratory infection	4	3 (12.73%)	1(1.89%)
Incomplete intestinal obstruction	3	2 (3.64%)	1(1.89%)
Abdominal infection	2	1 (1.82%)	1(1.89%)
Intestinal perforation + Intraperitoneal haemorrhage + Respiratory failure	1	0 (0%)	1(1.89%)

**Table 5:** Fourfold table of each complication according to the incidence of complications and the level of OPNI.

Complications	OPNI low group		OPNI high group	P
Intraperitoneal haemorrhage	Y	8	1	0.018
	N	47	52	
Wound infection	Y	4	2	0.430
	N	51	51	
Intestinal perforation or fistula	Y	3	2	0.679
	N	52	51	
Respiratory infection	Y	3	1	0.329
	N	52	52	
Incomplete intestinal obstruction	Y	2	1	0.582
	N	53	52	
Abdominal infection	Y	1	1	0.979
	N	54	52	
Intestinal perforation + intraperitoneal haemorrhage + Respiratory failure	Y	0	1	0.308
	N	55	52	
YES: Y NO: N				

**Figure 2:**
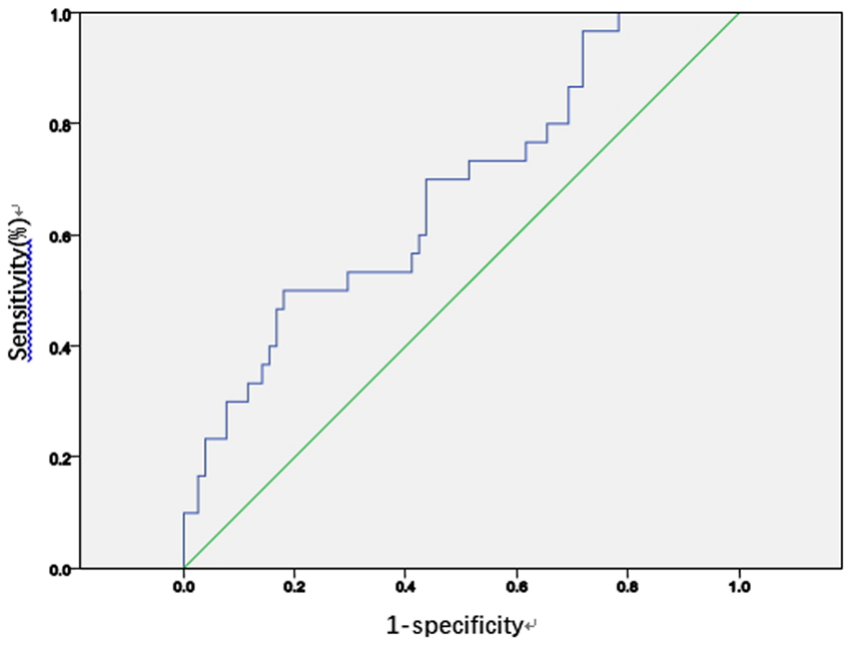
ROC curve of the NLR.

We drew the ROC curve of NLR according to whether postoperative complications occurred (Fig. 2).

The area under the curve was 0.675. When the NLR was 4.1, the Youden index was maximal, with a sensitivity of 70% and specificity of 56.4%, and the prediction of the risk of postoperative complications was most accurate. Taking 4.1 as the cutoff value, patients were divided into the high NLR group (NLR ≥ 4.1, n = 55) and the low NLR group (NLR < 4.1, n = 53). The differences in clinical features between the two groups were then analysed (Table 6).

**Table 6:** Clinical features of the high NLR group (n = 55) versus the low NLR group (n = 53).

clinical features	h-NLR G (53 cases)	l-NLR G (55 cases)	P	clinical features	h-NLR G (53 cases)	l-NLR G (55 cases)	P
**Gender**			>0.05	PL			>0.05
Male	31	36		L1+L2	23	27	
Female	24	17		L3	32	26	
**Age(y)**			>0.05	**DT**			**<0.05**
≤40(A1+A2)	31	31		B1+B2	31	41	
>40(A3)	24	22		B3	24	12	
**SH**			>0.05	**POD (y)**			>0.05
NO	37	33		≤1	29	31	
YES	18	20		1**<**n≤3	10	12	
**HA**			>0.05	3**<**n≤5	5	4	
NO	41	42		5**<**n≤10	10	5	
YES	14	11		>10	1	1	
**EO**			>0.05	**PNC**			**<0.05**
NO	41	40		Normal	42	50	
YES	14	13		Higher	13	3	
**BT**			>0.05	**PLC**			**<0.05**
A+	9	18		Normal	28	51	
B+	18	15		Lower	27	2	
O+	19	17		**PH**			>0.05
AB+	9	3		Normal	15	16	
**EM**			>0.05	Lower	40	37	
NO	42	39		**PPA**			>0.05
YES	13	14		Normal	10	18	
**PL**			>0.05	Lower	45	35	
NO	47	47		**PA**			>0.05
YES	8	6		Normal	21	28	
**p-BMI**			>0.05	Lower	34	25	
**<**18.5	38	31		**PEN**			>0.05
≥18.5	17	22		EN	27	35	
				NO EN	28	18	

**h-NLR G**, high NLR group; **l-NLR G**, low NLR group; **SH**, smoking history; **HA**, history of appendectomy; **POD**, preoperative duration; **PL**, primary lesion; **DT**, disease type (B3 or not); **EM**, extraintestinal manifestations; **PL**, perianal lesions; **EO**, emergency operation; **p-BMI**, preoperative BMI; **BT**, blood type; **PH**, preoperative haemoglobin; **PNC**, preoperative neutrophil count; **PLC**, preoperative lymphocyte count; **PA**, preoperative albumin; **PPA**, preoperative prealbumin; **PEN**, preoperative enteral nutrition.

Compared with the low NLR group, the high NLR group had a significantly greater incidence of: disease type B3 (X2 = 5.354, *P* = 0.021), preoperative neutrophilia (X2 = 5.560, *P* = 0.018), and preoperative lymphopenia (X2 = 25.961, *P* = 0.000).

In order to find what specific types of complications can be caused by high NLR, we calculated the incidence of seven complications in the low and high NLR group (Table 7) and each complication was listed as fourfold table according to the incidence of complications and the level of NLR (Table 8). we found that when NLR was higher than cut-off value (4.1), Crohn’s disease patients were more likely to develop lung infection (7.27%, P = 0.046).

**Table 7:** The incidence of seven complications in the low and high NLR group.

NLR	Total number	low group(n = 53 **<** 4.1) Number (Proportion)	high group(n = 55 > 4.1) Number (Proportion)
Complications			
Intraperitoneal haemorrhage	9	4(7.54%)	5(9.09%)
Wound infection	6	1(1.89%)	5(9.09%)
Intestinal perforation or fistula	5	2(3.77%)	3(5.45%)
Respiratory infection	4	0(0%)	4(7.27%)
Incomplete intestinal obstruction	3	1(1.89%)	2(3.64%)
Abdominal infection	2	0(0%)	1(1.82%)
Intestinal perforation + Intraperitoneal haemorrhage + Respiratory failure	1	1(1.89%)	0(0%)

**Table 8:** Fourfold table of each complication according to the incidence of complications and the level of NLR

Complications		NLR low group	NLR high group	P
Intraperitoneal haemorrhage	Y	4	5	0.773
	N	49	50	
Wound infection	Y	1	5	0.104
	N	52	50	
Intestinal perforation or fistula	Y	2	3	0.679
	N	51	52	
Respiratory infection	Y	0	4	0.046
	N	53	51	
Incomplete intestinal obstruction	Y	1	2	0.582
	N	52	53	
Abdominal infection	Y	0	1	0.326
	N	53	54	
Intestinal perforation + Intraperitoneal haemorrhage + Respiratory failure	Y	1	0	0.308
	N	52	55	
YES: Y NO: N				

### Correlation Between OPNI and NLR and Independent Risk Factors for Postoperative Complications

The OPNI and NLR were significantly negatively correlated (r = **−**0.420, *P* = 0.000, Table 9).

**Table 9:** Correlation analysis of OPNI and NLR.

			OPNI	NLR
Spearman’s rho	OPNI	correlation coefficient	1.000	−0.420
		Sig. (2-tailed)	.	0.000
		N	108	108
	NLR	correlation coefficient	−0.420	1.000
		Sig. (2-tailed)	0.000	.
		N	108	108

The OPNI and NLR were then combined to calculate the incidence of postoperative complications (Table 10). The incidence of postoperative complications in patients with concurrent low OPNI and high NLR was 48.6%, which was significantly higher than in patients with other OPNI and NLR combinations (X2 = 12.255, *P* = 0.007).

**Table 10:** Incidence of postoperative complications by combination of OPNI and NLR.

Combination	NO.	Complications	No complications	incidence	P
**l-OPNI G/l-NLR G**	20	5	15	25%	**0.007**
**h-OPNI G/l-NLR G**	33	4	29	12.1%	
**l-OPNI G/h-NLR G**	35	17	18	48.6%	
**h-OPNI G/h-NLR G**	20	4	16	20%	

**h-OPNI G**, high OPNI group; **l-OPNI G**, low OPNI group; **h-NLR G**, high NLR group; **l-NLR G**, low NLR group.

The OPNI and NLR were included into univariate analysis of risk factors for postoperative complications, then we screened meaningful variables (Table 11).

**Table 11:** Univariate analysis of risk factors for postoperative complications.

clinical features	Complications	No Complications	P	clinical features	Complications	No Complications	P
**Gender**			>0.05	**PL**			>0.05
Male	18	49		L1+L2	12	38	
Female	12	29		L3	18	40	
**Age(y)**			>0.05	**DT**			>0.05
≤40(A1 + A2)	20	42		B1 + B2	16	56	
>40(A3)	10	36		B3	14	22	
**SH**			**<0.05**	**POD(y)**			>0.05
NO	14	56		≤1	13	47	
YES	16	22		1 < n ≤ 3	7	15	
**HA**			>0.05	3 < n ≤ 5	4	5	
NO	20	63		5**<**n ≤ 10	6	9	
YES	10	15		>10	0	2	
**EO**			>0.05	**PNC**			>0.05
NO	23	58		Normal	24	68	
YES	7	20		Higher	6	10	
**BT**			>0.05	**PLC**			**<0.05**
A+	5	22		Normal	17	62	
B+	8	25		Lower	13	16	
O+	13	23		**PH**			>0.05
AB+	4	8		Normal	7	24	
**EM**			>0.05	Lower	23	54	
NO	26	55		**PPA**			**<0.05**
YES	4	23		Normal	3	25	
**PL**			>0.05	Lower	27	53	
NO	26	68		**PA**			>0.05
YES	4	10		Normal	9	40	
**p-BMI**			>0.05	Lower	21	38	
**<**18.5	16	53		**PEN**			**<0.05**
≥18.5	14	25		EN	11	51	
**OPNI**			**<0.05**	NO EN	19	27	
h-OPNI G	8	45		**NLR**			**<0.05**
l-OPNI G	22	33		h-NLR G	21	34	
				l-NLR G	9	44	

**SH**, smoking history; **HA**, History of appendectomy; **EM**, extraintestinal manifestations; **PL**, perianal lesions; **p-BMI**, preoperative BMI; h-OPNI G, high OPNI group; l-OPNI G, low OPNI group; **PL**, primary lesion; **DT**, disease type (B3 or not); **POD**, preoperative duration; **PNC**, preoperative neutrophil count; **PLC**, preoperative lymphocyte count; **PH**, preoperative haemoglobin; **PPA**, preoperative prealbumin; **PA**, preoperative albumin; **PEN**, preoperative enteral nutrition; h-NLR G, high NLR group; l-NLR G, low NLR group.

Univariate analysis showed that the following indicators were statistically significant: history of smoking within 1 year preoperatively (X2 = 5.999, *P* = 0.014); enteral nutritional intake within 2 weeks preoperatively was less than 500 kcal/day (X2 = 7.308, *P* = 0.007); lower than normal preoperative lymphocyte count (X2 = 5.745, *P* = 0.017) and prealbumin (X2 = 4.398, *P* = 0.036); low OPNI (X2 = 8.345, *P* = 0.004), and high NLR (X2 = 6.047, *P* = 0.014).

The above results were further analysed by logistic regression (Table 12). The independent risk factors for postoperative complications were: history of smoking within 1 year preoperatively (OR 3.006, 95% CI 1.170–7.727, *P* = 0.022), OPNI (OR 2.727, 95% CI 1.024–7.262, *P* = 0.045) and NLR (OR 2.782, 95% CI 1.042–7.425, *P* = 0.041).

**Table 12:** Logistic regression analysis of the incidence of postoperative complications.

Parameter	B	Standard Error	Wald	P	OR	95% Confidence interval, CI
**SH**	1.101	0.482	5.223	**0.022**	3.006	1.170–7.727
**OPNI**	1.003	0.500	4.030	**0.045**	2.727	1.024–7.262
**NLR**	1.023	0.501	4.173	**0.041**	2.782	1.042–7.425
**PLC**	0.348	0.583	0.356	0.551	1.416	0.452–4.443
**PPA**	0.909	0.704	1.668	0.197	2.481	0.625–9.857
**PEN**	0.438	0.551	0.632	0.427	1.775	0.569–5.533

**SH**, smoking history; **PLC**, preoperative lymphocyte count; **PPA**, preoperative prealbumin; **PEN**, preoperative enteral nutrition.

## Discussion

CD is a chronic non-specific intestinal inflammatory disease that tends to recur throughout the patient’s life. The incidence of CD shows significant distributional differences according to race and region. A 2013 study on the geographic variation and environmental risk factors for CD showed that the incidence of CD is higher in Europe and America, with an average annual prevalence of 13.7–198.5/10^5^ and a morbidity of 3.74–14.6/10^5^ ^7^. The prevalence and morbidity of CD in China are increasing each year; the prevalence and morbidity of CD were 2.29/10^5^ and 1.21/10^5^, respectively, from 2003 to 2007, which was increased compared with that from 1950 to 2002 (1.38/10^5^ and 0.28/10^5^, respectively)^8^.

The treatment of CD is mainly based on internal medicine; however, surgical intervention is still needed. In the natural course of CD, about 80% of patients need at least one surgical treatment in their lifetime^9^. The high incidence of postoperative complications in CD presents a challenge for surgeons. The incidence of postoperative complications in CD patients is significantly higher than after other intestinal resection surgery and is considered very satisfactory if the incidence is 10%^10, 11^. The incidence of postoperative complications in CD patients in China is 9.3–38%^12^. In the present study, postoperative complications occurred in 30 patients, and the overall incidence of postoperative complications was 27.8%, which is consistent with the literature. Death occurred in six patients. Therefore, it is very important to evaluate the risk factors of postoperative complications in CD patients.

OPNI is a tool based on nutritional and immune status to assess patients’ nutritional and inflammatory status and predict surgical risk. Albumin is synthesized by the liver, and albumin levels decline if nutritional intake is inadequate for a long period; hence, albumin can be used as an index of chronic protein malnutrition to reflect the general nutritional status of patients. The lymphocyte count reflects both the nutritional status and the immune function of patients. A decline in lymphocyte count can be caused by either malnutrition or poor cellular immune function^13^. Many scholars reported that the OPNI plays an important role in the prognosis evaluation of digestive system neoplasms.

CD is a chronic inflammatory and autoimmune disease that occurs as a result of abnormal immune function of T cells^14^. Most CD patients already have a malnutritional status preoperatively, which seriously affects postoperative recovery^15^. We designed this study to determine whether the OPNI could be used as a predictor of CD postoperative complications. In order to increase the scientific accuracy of the present study, we used the cutoff value calculated by the ROC curve to divide patients into the high and low OPNI groups, instead of using an empirical value (45) like in many previous studies.

Our study shows that the OPNI is an independent predictor of postoperative complications. Low OPNI indicates that the patient status was one of malnutrition and poor immunization. We also found that the high OPNI group had a greater incidence of non-B_3_ type CD than B_3_ type. According to clinical experience, the nutritional status of most B_3_ type patients is indeed worse than that of non-B3 patients, which suggests that disease type was a possible factor affecting the OPNI.

The enteral nutritional intake of most patients in the high OPNI group was more than 500 kcal/day within 2 weeks preoperatively, which suggests that the administration of enteral nutrition preoperatively was a possible factor affecting the OPNI. We can improve the prognosis of CD patients by increasing the OPNI by reasonable application of enteral nutrition. Compared with parenteral nutrition, enteral nutrition has its unique advantages in treatment of CD, such as regulating the intestinal tract flora, maintaining remission state, and promoting intestinal mucous rehabilitation^16^. CD recurrence can be effectively prevented if the energy provided by the elemental diet is more than 900 kcal/day^17^. This is further enhanced by the addition of specific nutrients, such as glutamine, omega-3 polyunsaturated fatty acids, and probiotics.

The early stage of inflammation is a proinflammatory state mediated by inflammatory factors such as TNF-α, interleukin-1, and interleukin-6, which are released by neutrophils, macrophages, and monocytes. This systemic inflammatory response is associated with the inhibition of neutrophil apoptosis, but it can also cause tissue damage^18^. Lymphocyte apoptosis in the spleen and thymus also increases correspondingly, causing immunosuppression, multiple organ dysfunction, and death^19^. Therefore, the NLR is an index for measuring systemic inflammation^20^. The influence of CD on inflammation, the application of immunosuppressive agents, and preoperative severe malnutrition result in an increase in neutrophils and a decrease in lymphocytes in CD patients. This increase in lymphocytes, decrease in neutrophils, and improvement in the systemic inflammatory response happen simultaneously^21^. More serious complications may occur if neutrophils rise and lymphocyte decline continuously for 1 week. Thus, the NLR can reflect the severity of the disease. In 2011, it was reported that the NLR is superior to white blood cell count in predicting the prognosis of acute pancreatitis^22^. In 2012, it was reported that the NLR is associated with the severity of non-alcoholic hepatic adipose infiltration^23^. In 2013, it was reported that the NLR could be used as a predictive index of the severity of ulcerative colitis^24^. However, the relationship between the NLR and CD has not been reported until now. Our findings suggest that the NLR of non-B_3_ patients is lower than that of B_3_ patients. Most non-B_3_ patients belonged to the low NLR group. According to clinical experience, the severity of B_3_ patients is indeed worse than that of non-B_3_ patients. Therefore, we consider that the NLR can predict the severity of CD, which is closely linked with prognosis. Univariate and multivariate analysis showed that when the NLR was ≥4.1, the risk of postoperative complications increased by 2.782 times.

The OPNI is a predictive index based on nutrition and immunity, and the NLR is an indicator for measuring systemic inflammation. We found that OPNI and NLR in CD patients were significantly negatively correlated. More severe systemic inflammation results in a worse nutrition status, and an increased risk of postoperative complications. The incidence of postoperative complications in patients with concurrent low OPNI and high NLR was 48.6%, which was much higher than the other groups.

Our study shows that history of smoking within 1 year preoperatively was an independent risk factor for postoperative complications. The incidence of postoperative complications in CD patients with a smoking history was three times higher than that in patients without a smoking history. Other studies have also suggested that smoking is an important risk factor^25, 26^ for postoperative complications such as respiratory^27^ and circulatory issues^28, 29^, wound infection^30^, and delayed healing^31^. In contrast, a recent study reported the opposite^32^. Further research is required to determine whether these diametrically opposite conclusions were associated with racial differences, the amount of nicotine, and the body’s tolerance to nicotine.

The albumin level and neutrophil and lymphocyte counts that are needed for the calculation of OPNI and NLR are included in preoperative routine blood testing, which is simple, fast, inexpensive, and available even in the most basic hospital. The above indices can increase the accuracy of prediction of postoperative complications in CD patients, while not increasing the patient’s economic burden and physical pain. The combined application of OPNI and NLR has better predictive value and is worth further promotion in clinical practice.

### Limitations

As a retrospective study, there are many shortcomings in this research, such as limited sample size, and bias caused by single center analysis, which all can cause some interference to the results of the study. Therefore, the conclusion of this study needs to be verified by a larger sample of retrospective studies or prospective studies.

## Materials and Methods

### General Information

We reviewed the clinical records of 108 CD patients who underwent bowel resection in Peking Union Medical College Hospital between 2004 and 2013. There were 67 (62%) males and 41 (38%) females, and the male-to-female ratio was 1.6:1. Average patient age was 37.6 ± 13.1 years (range 13–70 years). Average duration of hospitalization was 45.9 ± 24.9 days (range 2–124 days). Average preoperative BMI was 18.0 ± 3.4 kg/m^2^ (range 11.7–30.42 kg/m^2^). Thirty-eight patients (35.2%) had a history of smoking within 1 year preoperatively, and 25 (25.9%) had a history of appendectomy. No patients had a family history of CD. Postoperative complications occurred in 30 cases, with an overall incidence rate of 27.8%; intraperitoneal haemorrhage occurred in 9/30 cases (30%), wound infection in 6/30 cases (20%), intestinal perforation or fistula in 5/30 cases (16.7%), respiratory infection in 4/30 cases (13.3%), incomplete intestinal obstruction in 3/30 cases (10%), abdominal infection in 2/30 cases (6.7%), and intestinal perforation in 1/30 cases (3.3%) in the first 17 days postoperatively and rebleeding on the first day after reoperation. The case with intestinal perforation died due to respiratory failure at last.

As a retrospective research, all experimental protocols and all methods performed were approved by Ethics Review Board of Chinese Academy of Medical Sciences and Peking Union Medical College Hospital (*CAMS& PUMCH*), and Ethical Review Number is S-K302.

All patients in this study were treated and followed-up in our department. We got their permission to use their blood test results during treatment for research. All the information/image(s) in this article can be in an online open-access publication.

## Inclusion and Exclusion Criteria

**Inclusion criteria:**

- No history of bowel resection
- Bowel resection performed for CD
- Postoperative pathological diagnosis of CD

**Exclusion criteria:**

- History of previous bowel resection
- Decreased neutrophils, lymphocytes and albumin in peripheral blood within 1 week preoperatively
- No follow-up record

A final total of 108 cases were included.

### Calculation of OPNI and NLR

According to routine blood and biochemical testing conducted within 1 week preoperatively, the OPNI and the NLR were calculated as follows:

1. OPNI = albumin (g/L) +5× lymphocyte count (109/L)
2. NLR = neutrophil count (109/L)/lymphocyte count (109/L)

### Grouping Criteria

There were 18 parameters investigated, including sex, age, history of smoking within 1 year preoperatively, history of appendectomy, preoperative disease duration, primary lesion, disease type (B3 or not), extraintestinal manifestations, perianal lesions, emergency surgery, preoperative body mass index (BMI), blood type, preoperative haemoglobin, preoperative neutrophil count, preoperative lymphocyte count, preoperative albumin, preoperative prealbumin, and preoperative intake of enteral nutrition. The classification method of patient age, disease location and disease type was in accordance with the CD Montreal standards formulated at the International Congress of Gastroenterology in 2005 [7]. Age was divided into three groups: A1 (<17 years), A2 (17–40 years) and A3 (>40 years). Disease location was divided into four groups: type L1 (ileum), type L2 (colon), type L3 (ileocolon), and type L4 (upper gastrointestinal tract). The cases in which the lesions involved both the upper gastrointestinal tract and L1–L3 were classified as L1–L3. The disease type was divided into three groups: type B1 (non-obstruction, non-perforation), type B2 (obstruction), and type B3 (perforation). All patients were divided into the non-enteral nutrition group or the enteral nutrition group, with an enteral nutritional intake of 500 kcal/day within 2 weeks preoperatively used as the cut-off.

### Statistical analysis

The data were analysed using statistical software SPSS version 19(SPSS, Solutions Statistical Package for the Social Sciences, manufacturer’s name: International Business Machines Corporation, IBM. Armonk, New York, U.S.A). Enumeration data were analysed using Pearson’s chi-squared test or Fisher’s exact test. Measurement data were analysed by the independent samples *t*-test, Mann-Whitney U test and Kruskal-Wallis test.

The Youden index = sensitivity − (1− specificity), and was calculated by the receiver operating characteristic (ROC) curve.

The OPNI and NLR values with optimal sensitivity and specificity were regarded as the cutoff values according to which all patients were divided into high and low OPNI and NLR groups. We then analysed the distribution of characteristics and correlation between OPNI and NLR. We performed logistic regression analysis to investigate the relationship between OPNI, NLR, and occurrence of complications in CD patients.

### Data availability statement

The datasets analyzed during the current study are not publicly available due to intellectual property protection but are available from the corresponding author on reasonable request.
